# A Low Level of NaCl Stimulates Plant Growth by Improving Carbon and Sulfur Assimilation in *Arabidopsis thaliana*

**DOI:** 10.3390/plants10102138

**Published:** 2021-10-09

**Authors:** Li Hongqiao, Akiko Suyama, Namiki Mitani-Ueno, Ruediger Hell, Akiko Maruyama-Nakashita

**Affiliations:** 1 Department of Bioscience and Biotechnology, Faculty of Agriculture, Kyushu University, 744, Motooka, Nishi-ku, Fukuoka 819-0395, Japan; li.hongqiao.538@s.kyushu-u.ac.jp (L.H.); aksuyama@nm.beppu-u.ac.jp (A.S.); 2 Department of Food and Fermentation Sciences, Faculty of Food and Nutrition Sciences, Beppu University, 82, Kita-ishigaki, Beppu-shi, Oita 874-8501, Japan; 3 Institute of Plant Science and Resources, Okayama University, Chuo 2-20-1, Kurashiki 710-0046, Japan; namiki-m@rib.okayama-u.ac.jp; 4 Centre for Organismal Studies, Heidelberg University, Im Neuenheimer Feld 360, 69120 Heidelberg, Germany; ruediger.hell@cos.uni-heidelberg.de

**Keywords:** sodium, chloride, carbon, sulfur, plant growth

## Abstract

High-salinity stress represses plant growth by inhibiting various metabolic processes. In contrast to the well-studied mechanisms mediating tolerance to high levels of salt, the effects of low levels of salts have not been well studied. In this study, we examined the growth of *Arabidopsis thaliana* plants under different NaCl concentrations. Interestingly, both shoot and root biomass increased in the presence of 5 mM NaCl, whereas more than 10 mM NaCl decreased plant biomass. To clarify the biological mechanism by which a low level of NaCl stimulated plant growth, we analyzed element accumulation in plants grown under different NaCl concentrations. In addition to the Na and Cl contents, C, S, Zn, and Cu contents were increased under 5 mM NaCl in shoots; this was not observed at higher NaCl concentrations. Adverse effects of high salinity, such as decreased levels of nitrate, phosphate, sulfate, and some cations, did not occur in the presence of 5 mM NaCl. An increase in C was possibly attributed to increased photosynthesis supported by Cl, Zn, and Cu, which also increased in shoots after NaCl application. Salt stress-responsive gene expression was enhanced under 20 mM NaCl but not at lower doses. Among the S metabolites analyzed, cysteine (Cys) was increased by 5 mM NaCl, suggesting that S assimilation was promoted by this dose of NaCl. These results indicate the usefulness of NaCl for plant growth stimulation.

## 1. Introduction

High salinity has become an important issue in agricultural production in many regions of the world and has led to a drastic reduction in plant growth and biomass production of crops [1,2,3]. Under high salinity, which usually corresponds to a high concentration of NaCl in the soil environment, the cellular concentrations of sodium (Na^+^) and chloride (Cl^−^) ions increase. This causes osmotic stress and leads to toxic effects, including a decrease in the level of other cations, such as K^+^, production of reactive oxygen species (ROS), and inhibition of membrane integrity, protein synthesis, cellular metabolism, photosynthesis, and water and nutrient acquisition [2,4]. To cope with these toxic effects of NaCl, plants have evolved the salt overly sensitive (SOS) pathway, which regulates ion homeostasis under salinity stress [2,4,5].

Because of its severe impact on agricultural production, research pertaining to understanding the effects of NaCl is mainly focused on analyzing the effects of high doses (more than 50 mM), while the potential beneficial effects of low doses are relatively less investigated. Sodium is a beneficial element for plants [6,7]. Na functions as an indispensable element for most C_4_ species, as Na^+^ is required for pyruvate import to the chloroplast, which supports the carbon fixation process in C_4_ photosynthesis [7,8]. Nitrogen uptake and assimilation, as well as the activity of photosystem II, require Na in C_4_ species [7]. In addition to C_4_ plants or halophytes, many C_3_ plants have shown growth stimulation upon Na application [6,7]. Under potassium (K) deficiency, Na can partly substitute the functions of K, e.g., by serving as an osmoticum, a counter ion for anion in long-distance transport, and an enzymatic activator [7,9,10]. Growth stimulation by a relatively low level of NaCl (less than 20 mM) has also been reported for Chenopodiaceae, sugar beet, red beet, tomato, potato, sunflower, etc., even under an adequate supply of K [7,11]. Improvement of water use efficiency was observed in Chrysanthemum with 50 mM NaCl [12]. However, growth promotion by a low level of NaCl largely depends on the plant species and on growth conditions [7].

Chlorine is an essential nutrient for plants and is involved in osmoregulation and turgor maintenance [6]. In the chloroplast, Cl^−^ functions as an indispensable component of photosystem II (PSII) by stabilizing the water de-composition/oxygen liberation system [13,14]. In addition, Cl^−^ regulates the activities of some enzymes, such as asparagine synthetase, vacuolar proton pump ATPase, and amylase [13,15]. The regulation of Cl^−^ homeostasis is critical in stomatal guard cells and growing pollen tubes [16]. Various kinds of crops respond significantly to Cl^−^ fertilizers [11,17,18], although most studies have not clarified the effects of accompanying cations (such as Na^+^) on the increased agricultural yield [14]. Low concentrations of Cl^−^ (1 to 5 mM) promote plant growth by increasing leaf cell size, reducing transpiration, and improving water-use efficiency in tobacco and tomato plants [19,20].

The relationship between sulfur (S) metabolism and salt has also been mainly investigated with respect to the contributions of S assimilation and S-containing compounds to salt stress. Plants take up S mostly as sulfate through the activity of sulfate transporters (SULTR). Intracellular sulfate is activated to adenosine 5′-phosphosulfate (APS) by ATP sulfurylase and then reduced to sulfide by the actions of APS reductase (APR) and sulfite reductase. Then, sulfide is converted to cysteine (Cys) upon reaction with *O*-acetyl-L-serine, which is catalyzed by *O*-acetyl-L-serine (thiol) lyase [21,22,23]. The common antioxidant glutathione (GSH) is synthesized from Cys via ATP-dependent reactions catalyzed by gamma-glutamylcysteine synthase and GSH synthase. Under salt stress, various plants, including *Arabidopsis*, induce Cys biosynthesis to prevent oxidative stress caused by high salinity [24,25]. Indeed, the overexpression of enzymes involved in Cys and GSH synthesis increases the salt tolerance of plants [26,27]. However, the contribution of S assimilation to growth stimulation caused by low NaCl concentrations has not been analyzed till date.

Based on this background, we aimed to demonstrate the effect of NaCl dosage on plant growth, element accumulation, and S assimilation in the model plant *Arabidopsis thaliana.* We found that a low level of NaCl stimulates plant growth. The analysis of elements and S metabolites suggested that increased S assimilation and content of some elements could contributed to enhanced plant growth under low levels of NaCl.

## 2. Results

### 2.1. Low Concentration of NaCl Promoted Plant Growth

**Figure 1:**
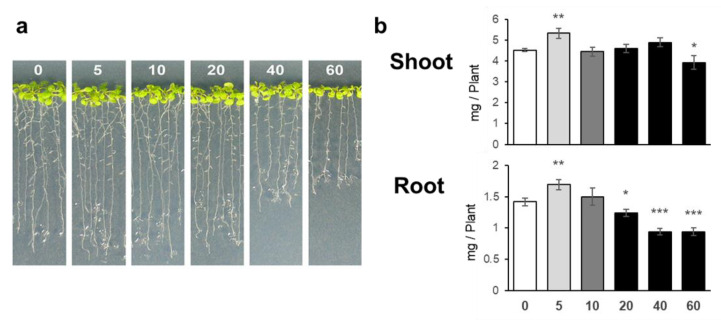
Plant growth under NaCl treatment. Plants were grown for 11 days on agar medium containing 0, 5, 10, 20, 40, or 60 mM NaCl. NaCl concentrations are indicated by the number on each photo. After capturing the images of the plants (**a**), shoots and roots were harvested separately. (**b**) Fresh weight of shoots (upper) and roots (lower). Bars and error bars represent the mean and standard error (*n* = 6), respectively. Asterisks indicate significant differences between NaCl-treated and non-treated plants as calculated using Student’s *t*-test (* 0.01 ≤ *p* < 0.05, ** 0.001 ≤ *p* < 0.01, *** *p* < 0.001).

Plants were grown for 11 days on agar medium containing 0, 5, 10, 20, 40, or 60 mM NaCl (Figure 1a). The shoot and root fresh weights under 5 mM NaCl were significantly higher than those without NaCl application, whereas root fresh weights decreased in the presence of 20 mM and higher concentrations of NaCl (Figure 1b). These results indicated that the presence of 5 mM NaCl positively influenced plant growth on the agar medium, which was in clear contrast with the obvious growth retardation observed in the presence of 20 mM and higher concentrations of NaCl. As we considered appealing the growth stimulation by 5 mM NaCl, we focused on lower doses of NaCl in the following analysis.

### 2.2. Increase in NaCl Concentration Decreased Nitrate and Sulfate Content, but Their Levels Were Maintained under 5 mM NaCl

**Figure 2:**
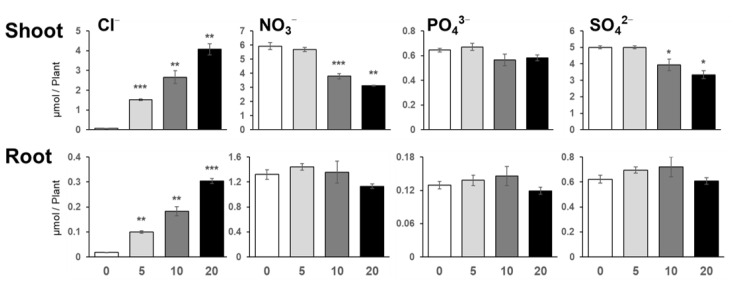
Chloride (Cl^−^), Nitrate (NO_3_^−^), Phosphate (PO_4_^3−^), and Sulfate (SO_4_^2−^) contents in plants under NaCl treatment. Cl^−^, NO_3_^−^, PO_4_^3−^, and SO_4_^2−^ contents were analyzed in plants grown for 11 days on agar medium containing 0, 5, 10, or 20 mM NaCl, using ion chromatography. Bars and error bars represent the mean and standard error (*n* = 4). Asterisks indicate significant differences between the NaCl-treated and the non-treated plants as calculated using Student’s *t*-test. (* 0.01 ≤ *p* < 0.05, ** 0.001 ≤ *p* < 0.01, *** *p* < 0.001).

To determine the effects of NaCl application on the content of chloride (Cl^−^) and other anions in plants, we analyzed Cl^−^, nitrate (NO_3_^−^), phosphate (PO_4_^3−^), and sulfate (SO_4_^2−^) contents using ion chromatography (Figure 2). The application of NaCl resulted in a significant increase in Cl^−^ content in both the shoots and the roots of *Arabidopsis*. Cl^−^ levels increased in both shoots and roots even under 5 mM NaCl and further continuously increased with increasing NaCl concentration. NO_3_^−^ and SO_4_^2−^ contents in shoots and roots were maintained and not affected by 5 mM and 10 mM NaCl; however, the contents of these ion started to decrease in shoots when higher concentrations of NaCl were added to the growth medium. NO_3_^−^ and SO_4_^2−^ contents in roots and PO_4_^3−^ content in both shoots and roots were not influenced by these NaCl doses.

### 2.3. Effects of NaCl on Elemental Contents

**Figure 3:**
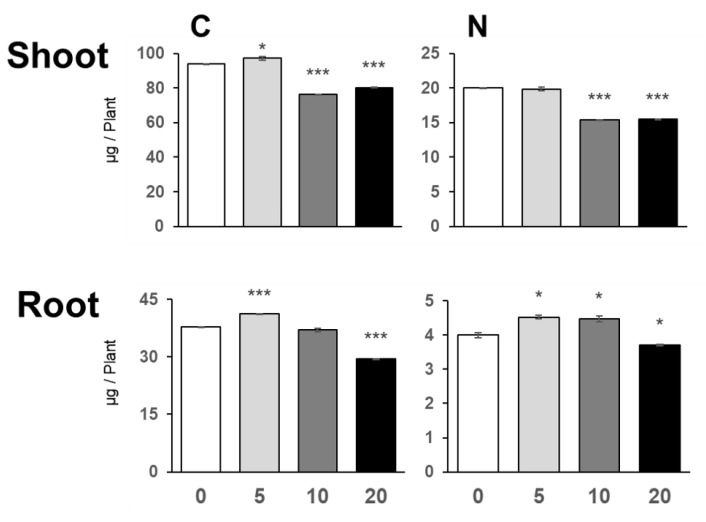
Carbon and Nitrogen contents in plants under NaCl treatment. C and N contents in plants grown for 11 days on agar medium containing 0, 5, 10, or 20 mM NaCl were analyzed with an elemental analyzer. Bars and error bars represent the mean and standard error (*n* = 3). Asterisks indicate significant differences between the NaCl-treated and the non-treated plants as calculated using Student’s *t*-test (* 0.01 ≤ *p* < 0.05, ** 0.001 ≤ *p* < 0.01, *** *p* < 0.001).

To understand how low levels of NaCl stimulate plant growth, we analyzed the elemental contents in plants grown under 0, 5, 10, and 20 mM NaCl (Figure 3). Carbon content was significantly increased by 5 mM NaCl in both shoots and roots but was decreased by 10 and 20 mM NaCl. Nitrogen content in roots was increased by 5 and 10 mM NaCl but was reduced by 20 mM NaCl. In shoots, 5 mM NaCl did not change the N content, but a higher NaCl concentration significantly reduced it. These findings may point to enhanced biomass production with respect to C and N assimilation at low NaCl concentrations.

**Figure 4:**
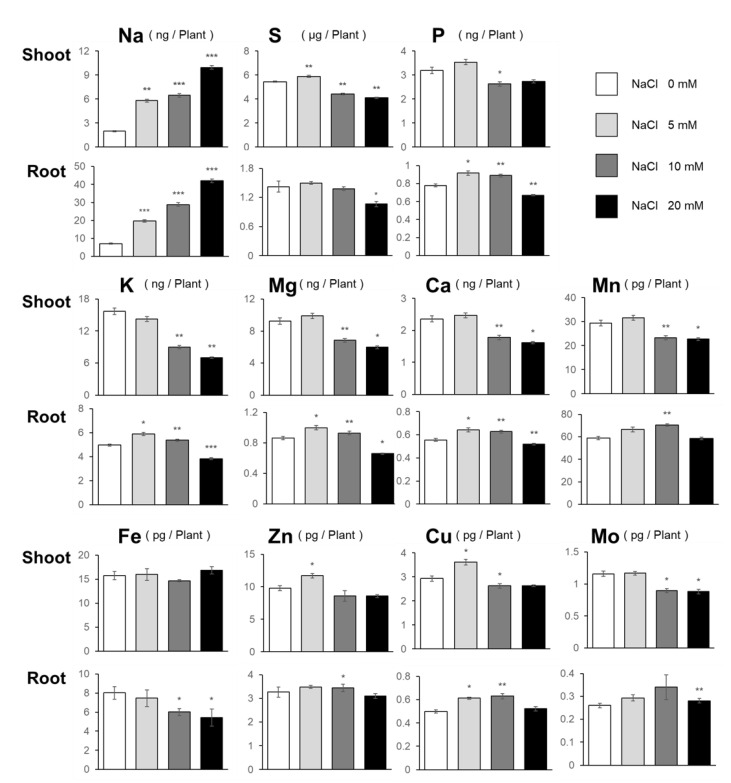
Sodium and essential elements’ content in plants under NaCl treatment. Na, Sulfur, Phosphorus, Potassium, Magnesium, Calcium, Manganese, Iron, Zinc, Copper, and Molybdenum contents in plants grown for 11 days on agar medium containing 0, 5, 10, or 20 mM NaCl were measured using ICP–MS. Bars and error bars represent the mean and standard error (*n* = 3). Asterisks indicate significant differences between the NaCl-treated and the non-treated plants as calculated using Student’s *t*-test. (* 0.01 ≤ *p* < 0.05, ** 0.001 ≤ *p* < 0.01, *** *p* < 0.001).

In addition to total C and N, we analyzed the content of essential elements, viz., P, S, K, Mg, Ca, Mn, Fe, Zn, Cu, and Mo, along with that of Na, in NaCl-treated plants (Figure 4). Na content increased with increasing NaCl concentration and was consistently higher in roots than in shoots. In addition to Na, 5 mM NaCl in the growth medium increased S, Zn, and Cu contents in shoots, and P, K, Mg, Ca, and Cu contents in roots. In contrast, the presence of 10 and 20 mM NaCl resulted in a decrease in most of the elements, including S, K, Mg, Ca, Mn, Cu, and Mo, in shoots. In roots, the presence of 10 mM NaCl led to an increase in the levels of some elements, such as P, K, Mg, Ca, Mn, Zn, and Cu, but 20 mM NaCl decreased most of the elements except for Mn, Zn, and Cu. Mo content in the roots was slightly increased under 20 mM NaCl. These results indicated that 5 mM NaCl increased the content of various elements.

### 2.4. Low Level of NaCl Increased Cys Content in Shoots

**Figure 5:**
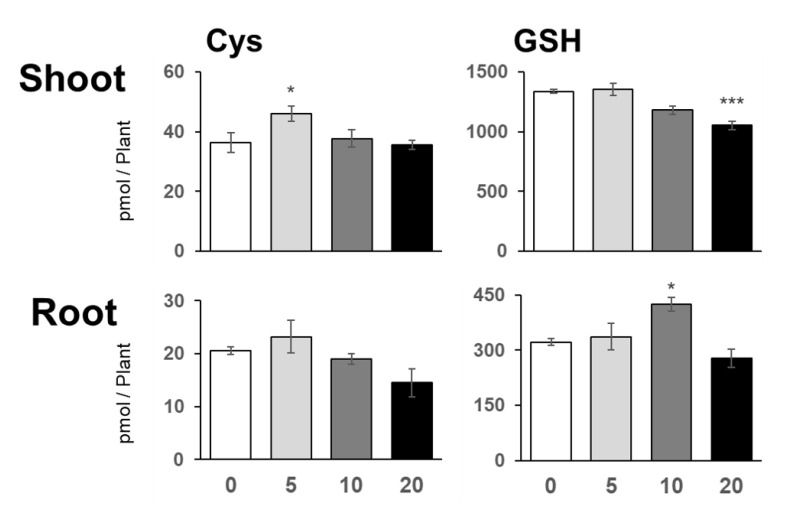
Cysteine (Cys) and Glutathione (GSH) contents in plants under NaCl treatment. Cys and GSH contents in plants grown for 11 days on agar medium containing 0, 5, 10, or 20 mM NaCl were analyzed using an HPLC fluorescence detector system. Bars and error bars represent the mean and standard error (*n* = 4). Asterisks indicate significant differences between the NaCl-treated and the non-treated plants as calculated using Student’s *t*-test (* 0.01 ≤ *p* < 0.05, ** 0.001 ≤ *p* < 0.01, *** *p* < 0.001).

Plant growth and elemental analysis showed that low-NaCl-dependent plant growth stimulation correlated with increased C, S, Zn, and Cu levels in shoots. Increased C levels may be attributed to photosynthesis, as supported by the increased Cl, Mn, Cu, and Mg contents [28]. Here, we focused on the increased S content to demonstrate how it may contribute to increased plant growth and examined the contents of the major S compounds, namely, Cys and GSH (Figure 5). 

Cys content in shoots increased under 5 mM NaCl, and a similar tendency was observed in root Cys content (Figure 5). The application of higher levels of NaCl did not affect the Cys content in either shoots or roots. GSH content in shoots was reduced in the presence of 20 mM NaCl; however, that in roots was increased by 10 mM NaCl and was maintained by 5 mM NaCl.

**Figure 6:**
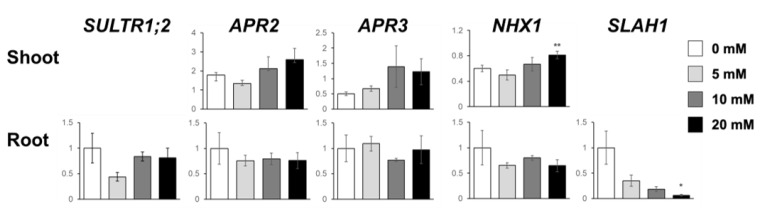
Expressions of S assimilatory and salt stress-responsive genes in plants under NaCl treatment. Gene expression of *SULTR1;2, APR2, APR3, NHX1,* and *SLAH1* was analyzed by quantitative RT-PCR. Relative mRNA abundance was determined using the ΔΔCt method, and *ubiquitin* (*UBQ2*, accession no. J05508) was used as an internal control. Values relative to the roots without NaCl treatment are presented. Asterisks indicate significant differences between the NaCl-treated and the non-treated plants as calculated using Student’s *t*-test (* 0.01 ≤ *p* < 0.05, ** 0.001 ≤ *p* < 0.01, *** *p* < 0.001).

Since S and Cys levels were increased by 5 mM NaCl, we analyzed the expression of some S assimilatory genes, namely, *SULTR1*;*1*, *SULTR1*;*2*, *APR2*, and *APR3*, which are involved in reactions known as key processes in S assimilation [21]. To know whether plants sensed 5 mM NaCl as salt stress, we also analyzed the expression of the salt stress-responsive genes *NHX1* and *SLAH1* [2] (Figure 6). As *SULTR1;1* expression was too low to be detected, we present the transcript levels of *SULTR1;2*, *APR2*, and *APR3*. The expression of these genes did not increase with these NaCl dosages (Figure 6). The transcript levels of *NHX1* and *SLAH1* responded to 20 mM NaCl but not to 5 and 10 mM NaCl, i.e., 20 mM NaCl upregulated *NHX1* in shoots and downregulated *SLAH1* in roots (Figure 6).

### 2.5. Low Levels of KCl and NaNO_3_ Stimulated Plant Growth

**Figure 7:**
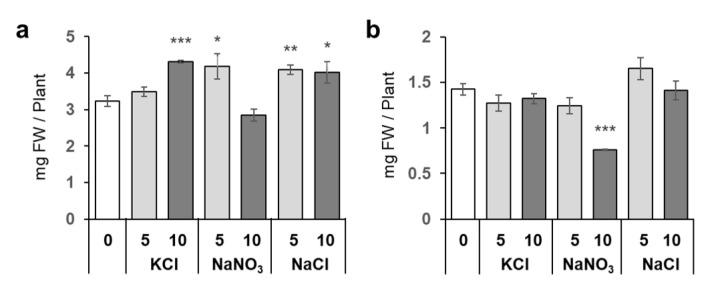
Plant growth in the presence of different salts. Plants were grown for 10 days on agar medium with or without 5 and 10 mM KCl, NaNO_3_, and NaCl. Shoot (**a**) and root (**b**) tissues were harvested separately, and fresh weights (FW) were recorded. Bars and error bars represent the mean and standard error (*n* = 4). Asterisks indicate significant differences between the salt–treated and the untreated plants as calculated using Student’s *t*-test (* 0.01 ≤ *p* < 0.05, ** 0.001 ≤ *p* < 0.01, *** *p* < 0.001).

To understand whether Na^+^ or Cl^−^ plays any crucial role in the growth stimulation of *Arabidopsis*, we tested plant growth in the presence of three different salts: KCl, NaNO_3_, and NaCl (Figure 7). Although the effective concentrations were different, both KCl (10 mM) and NaNO_3_ (5 mM) increased the fresh weight (FW) of plant shoots, as was observed with NaCl application. We found that 10 mM NaNO_3_ decreased the root FW, suggesting that this combination of increased Na^+^ and NO_3_^−^ could be detrimental for plants. These results indicated that both Na^+^ and Cl^−^ in this concentration range could increase plant growth by acting synergistically.

## 3. Discussion

### 3.1. Plant Growth Stimulation by Low-Level NaCl Is Accompanied by Increased C and S Levels

NaCl is known to be toxic when it is accumulated in the roots, and there are many studies on how plants respond to and tolerate high NaCl stress [2]. Under our study conditions, 20 mM and higher concentrations of NaCl decreased plant growth, but surprisingly, 5 mM NaCl stimulated plant growth (Figure 1 and Figure 7). At 5 mM NaCl, the Cl^−^ content in plants increased significantly, while the content of other anions, i.e., NO_3_^−^, PO_4_^3−^, and SO_4_^2−^, was not changed; however, 10 and 20 mM NaCl evidently inhibited these anions’ accumulation in shoots (Figure 2). Moreover, 5 mM NaCl increased the levels of C, S, Zn, and Cu in the shoots (Figure 3 and Figure 4). We also observed a significant increase in Cys levels, indicating that low levels of NaCl stimulated the S assimilation process (Figure 5). Expression of salt stress-responsive genes significantly increased only in those plants grown in the presence of 20 mM NaCl (Figure 6). These results suggest that more than 10 or 20 mM NaCl induces salt stress in plants; however, 5 mM NaCl does not induce such stress and, in fact, positively influences plant metabolism, resulting in plant growth.

The positive effects of low levels of NaCl seemed to be due to both Na^+^ and Cl^−^, as both KCl and NaNO_3_ stimulated plant growth (Figure 7). However, the salt concentrations that increased plant growth differed for KCl and NaNO_3_; 10 mM KCl promoted plant growth but not 5 mM KCl; 5 mM NaNO_3_ stimulated plant growth, whereas 10 mM NaNO_3_ inhibited plant growth. This suggests that the doses of Na^+^ and Cl^−^ capable of stimulating plant growth might be different, i.e., 10 mM for Cl^−^ and 5 mM for Na^+^ may promote plant growth; therefore, growth stimulation by 5 mM NaCl can be a result of combinatorial effects of both Na^+^ and Cl^−^. In the MGRL medium used in this study [29] (Supplemental Tables S1 and S2), the initial concentration of Na^+^ was 2.13 mM, and that of Cl^−^ was 24.8 µM, comparable to those in Hoagland’s medium containing 16 to 54 µM Na^+^ and 50 µM Cl^−^ [30] (Supplemental Table S4).

Although the deficiency threshold for Cl^−^ is accepted to be less than 100 µM, recent studies have demonstrated that some plants such as tobacco and tomato grew better with up to 5 mM Cl^−^ [14], indicating that MGRL and Hoagland’s media contain very low Cl^−^ levels compared to those supporting maximum plant growth. The shoot Cl^−^ concentrations analyzed in this study were 2.1, 53.9, 94.3, and 144.3 µg/plant under 0, 5, 10, and 20 mM NaCl application, respectively (Figure 2), which corresponded to 7.7, 183, 421, and 609 mg/g DW, respectively. Considering that Cl^−^ concentration in the shoots of non-halophyte plants ranges from 1 to 20 mg/g DW and that tobacco plants accumulate up to 50 mg/g DW when treated with 5 mM Cl^−^ [13,19,31], plant growth can be improved by increasing Cl^−^ concentration in the medium up till 5 mM. Although Na^+^ is not an essential element for plants, the application of a low amount of Na^+^ stimulated plant growth [6]. Even if high salinity is known to decrease the root-to-shoot transport of NO_3_^−^, PO_4_^3−^, and SO_4_^2−^ by competition with Cl^−^ (Figure 2) [13], plants in this study managed to maintain adequate levels of Cl^−^ under 5 mM NaCl, which resulted in adequate photosynthetic activity and osmotic conditions. 

### 3.2. How Does Low-Level NaCl Increase C and S Levels?

Growth stimulation by 5 mM NaCl was accompanied by increased C and S levels (Figure 3 and Figure 4). Since Cl^−^ supports the light reaction of photosynthesis, this concentration of Cl^−^ may stimulate photosynthesis and an eventual increase in the plant biomass (Figure 1) [13,14]. Consistently, the level of Cu, another element involved in photosynthesis, increased, and those of other elements such as Mg and Mn tended to increase under the same conditions (Figure 4). Another possibility is that NaCl application may stimulate sucrose uptake from the medium. Although the NaCl concentration used in this study was different from the high levels of NaCl in other studies, salinity stress is known to increase the sugar content in leaves and stems and as well as the expression of some sugar transporters [32,33]. Moreover, NaCl is known to stimulate sucrose transport into vacuoles and sucrose accumulation in storage tissues, possibly by stimulating ATPase activity at the tonoplast membrane in beet [7]. As C levels increased in both shoots and roots, increased photosynthesis and increased sucrose uptake and transport to vacuoles could contribute to the increased levels of C in the presence of a low concentration of NaCl.

Under salt stress, wherein the levels of S-containing metabolites such as Cys, GSH, and glucosinolates are increased [25,34,35], the increase in S metabolites is considered to improve the antioxidant capacity of plants. A high dose of NaCl (150–200 mM) increased the transcript levels and the activity of the enzymes involved in S assimilation, including ATP sulfurylase, *APR*, and *O*-acetylserine (thiol) lyase in *Arabidopsis* and *Brassica* crops [34,35,36,37]. In *Brassica rapa*, the expression of *SULTR1s* and sulfite reductase gene was decreased, but that of *SULTR4* was increased by 50 and 100 mM NaCl without affecting the total S level [38]. In our gene expression analysis, NaCl application did not influence the expression of S assimilatory genes, such as *SULTR1;2*, *APR2*, and *APR3* (Figure 6). These results suggested that the increased S level was not due to increased gene expression, although we did not analyze all S assimilatory genes. To maintain the cell’s ion balance, cation influx into the cell is accompanied by that of inorganic anions such as PO_4_^3−^, Cl^−^, NO_3_^−^, and SO_4_^2−^ [7]. This may happen when Na^+^ influx increases in roots, especially when Cl^−^ is easily distributed from root to shoot, consistent with the increased tendency of NO_3_^−^, PO_4_^3−^, and SO_4_^2−^ and the increase of N and P in 5 mM NaCl-treated roots (Figure 2). To explore this hypothesis, we need to investigate further how a low level of NaCl increases S assimilation.

### 3.3. Possible Contribution of Increased Zn and Cu Levels to the Growth Stimulation Caused by a Low Level of NaCl

Unlike the responses to high salinity, the decrease in Mg, Ca, and K levels due to competitive transport with Na^+^ [4,5] was not observed in the presence of 5 mM NaCl (Figure 4) [38]. Instead, there was a slight increase in Zn and Cu in the shoots and of Mg, Ca, and Cu in the roots (Figure 4).

Zn and Cu are required for photochemical processes involved in photosynthesis [39,40,41,42]. As metal cofactors, Zn and Cu are the components of the copper–zinc superoxide dismutase (Cu/Zn-SOD), which catalyzes the formation of H_2_O_2_ from superoxide radicals [42,43]. Overexpression of Cu/Zn-SOD significantly enhanced NaCl tolerance in *Arabidopsis* [44]. In addition to SOD, Cu acts as a cofactor of catalase and enzymes involved in the synthesis of phenolic compounds, which also function against oxidative damage in plants [42,43]. The plant growth stimulation caused by 5 mM NaCl (Figure 1) could be due to the increased photosynthetic activity supported by increased Cu and Zn contents in shoots and/or the increased antioxidant activity of SOD.

In this study, we found positive effects of low levels of NaCl on the growth of *Arabidopsis*. The elemental contents suggested that low levels of NaCl may provide a better ion balance among the elements, without disturbing cellular metabolism and the root-to-shoot distribution of other essential nutrients, and may even stimulate C and S assimilation, supporting plant growth and antioxidant activity. Although the precise mechanism has not been clarified, this finding can be implemented to provide better plant growth conditions.

## 4. Materials and Methods

### 4.1. Plant Materials and Growth Conditions

*Arabidopsis thaliana* plants, ecotype “Columbia” (Col-0), were used as the plant material. Plants were grown on mineral nutrient medium (Supplemental Tables S1 and S2) [29] containing 1% sucrose and 0.8% agarose at 22 °C under constant illumination (40 µmol m^−2^ s^−1^). For NaCl treatment, plants were vertically grown on medium supplemented with 0, 5, 10, 20, 40, or 60 mM NaCl (Sodium Chloride; Nacalai Tesque, Kyoto, Japan). Treatments with KCl (Potassium Chloride; Nacalai Tesque) and NaNO_3_ (Sodium Nitrate; Nacalai Tesque) were performed similarly.

### 4.2. Measurement of Fresh and Dry Weights

Eleven-day-old plants were separately harvested to obtain shoot and root tissues. After rinsing with distilled water, we counted the number of plants and determined their fresh weight (FW).

### 4.3. Anion Analysis

Plant tissues were frozen in liquid nitrogen, homogenized with the Tissue Lyser MM300 (Retsch, Haan, Germany), and extracted with 5 volumes of ultrapure water. The resultant mixtures were centrifuged at 4 °C, 16,150× *g* for 15 min. The supernatant was used for anion analysis using ion chromatography (IC-2001, TOSOH, Tokyo, Japan) as described previously [45,46]. Anion mixture standard solution 1 (Wako Pure Chemicals, Tokyo, Japan) was used as a standard. 

### 4.4. Elemental Analysis

Dried plant tissues were ground with the Tissue Lyser MM300 (Retsch, Germany) into fine powder. Carbon (C) and nitrogen (N) contents were analyzed in 1 mg of powder using CHN Corder MT-6 (Yanaco Apparatus Development Laboratory, Tokyo, Japan). The content of sulfur (S) and other elements was analyzed using inductively coupled plasma–mass spectroscopy (ICP–MS; Agilent7700X, Agilent Technologies, Santa Clara, CA, USA). We digested 1 mg of powder in 200 µL HNO_3_ at 95 °C for 30 min and then at 115 °C for about 90 min. The digested samples were diluted to 1 mL with extra-pure water and filtered using 0.45 µm filters (DISMIC-03CP, ADVANTEC, Tokyo, Japan). The filtered samples were diluted 10 times with a solution consisting of 0.1 M HNO_3_ and 10 µg L^−1^ gallium (KANTO CHEMICAL, Tokyo, Japan) as an internal standard before being subjected to ICP–MS. Quantification was performed using a standard curve obtained via serial dilutions of the standard solutions (KANTO CHEMICAL).

### 4.5. Analysis of Cysteine (Cys) and Glutathione (GSH)

The content of Cys and GSH was determined by an HPLC fluorescence detection system after labeling the thiol bases by monobromobimane, as described previously [47]. The labeled products were separated by HPLC using the TSKgel ODS-120T column (150 × 4.6 mm, TOSOH) and detected with a scanning fluorescence detector FP-920 (JASCO, Oklahoma, OK, USA), monitoring for fluorescence of thiol-bimane adducts at 478 nm under excitation at 390 nm. Cys and GSH (Nacalai Tesque) were used as standards.

### 4.6. Gene Expression Analysis

Total RNA was extracted from the shoot and root tissues using Sepasol-RNA I (Nacalai Tesque), and reverse transcription was conducted using the PrimeScript RT Reagent Kit with gDNA Eraser (Takara, Maebashi, Japan). Quantitative PCR was conducted using the KAPA SYBR FAST qPCR Master Mix 2× (Kapa Biosystems, Boston, MA, USA) and qTOWER3G touch (Analytik Jena AG, Germany). Relative mRNA abundance was determined using the ΔΔCt method, and *ubiquitin* (*UBQ2*, accession no. J05508) was used as a constitutive internal control. Gene specific primers for quantitative PCR are listed in Supplemental Table S3 [48,49,50].

### 4.7. Statistical Analysis

Statistical analyses were performed by the Student’s *t*-test using Microsoft Excel. The detected significant differences are indicated by asterisks (* 0.01 ≤ *p* < 0.05, ** 0.001 ≤ *p* < 0.01, *** *p* < 0.001).

## Data Availability

All data were provided within the article and Supplemental files.

## Supplementary Materials

The following are available online at https://www.mdpi.com/article/10.3390/plants10102138/s1, Table S1: Salt concentrations in the liquid culture medium, Table S2: Element concentrations in the liquid culture medium, Table S3: Primers used for quantitative RT-PCR, Table S4: Salt composition of Hoagland’s medium.
