# Supplementary material for: A Low Level of NaCl Stimulates Plant Growth by Improving Carbon and Sulfur Assimilation in Arabidopsis thaliana
Source: Plants (Basel). 2021 Oct 9;10(10):2138. doi: 10.3390/plants10102138 (PMC8541631; doi:10.3390/plants10102138)
Supplement: Supplementary file 1 [file plants-10-02138-s001.zip › plants-1399019-supplementary.pdf]

Supplemental Table S1 Salt concentrations in the liquid culture medium

Compound	Molecular Weight	Concentration in the stock solution (x 200)		Concentration in the medium
		mM	g/L	mM
$NaH_2PO_4 \cdot 2H_2O$	156.01	300	46.8	1.5
$Na_2HPO_4 \cdot 12H_2O$	358.14	50	17.9	0.25
$Ca(NO_3)_2 \cdot 4H_2O$	236.15	400	94.5	2
KNO_3	101.1	600	60.7	3
$MgSO_4 \cdot 7H_2O$	246.47	300	74.0	1.5
$Na_2 \cdot EDTA \cdot 2H_2O$	372.24	11.7	4.35	0.0585
$NaFeEDTA \cdot 3H_2O$ (FeIII)	385.06	1.9	0.732	0.00950
$MnCl_2 \cdot 4H_2O$	197.9	2.06	0.408	0.0103
$ZnCl_2$	136.29	0.202	0.0275	0.00101
$CuCl_2 \cdot 2H_2O$	170.48	0.192	0.0327	0.000960
H_3BO_3	61.83	6	0.371	0.0300
$(NH_4)_6Mo_7O_24 \cdot 4H_2O$	1235.86	0.0048	0.00593	0.0000240
$CoCl_2 \cdot 6H_2O$	237.93	0.026	0.00619	0.000130

Supplemental Table S2
Element concentrations in the liquid culture medium

Element	Concentration in the stock solution (x 200)	Concentration in the medium
	mM	mM
K	600	3
Na	423	2.12
Ca	400	2
P	350	1.75
S	300	1.5
N	2000	10
Mg	300	1.5
B	6	0.03
Cl	4.96	0.0248
Mn	2.06	0.0103
Fe	1.74	0.00870
Zn	0.202	0.00101
Cu	0.192	0.000959
Mo	0.0336	0.000168
Co	0.0260	0.000130

Supplemental Table S3 Primers used for quantitative RT-PCR

Category	Gene name	AGI	Forward primer 5'--3'		Reverse primer 5'--3'		Reference
Salt stress response	*NHX1*	AT5G27150	NHX1 _qF	CTACCTATTACCGCACCAGAACG	NHX1 _qR	CTCAATGAACGAGTCTTGGTCC	48
	SLAH1	AT1G62280	SLAH1 _qF	TCTTCATGTCCCTGGTCTG	SLAH1 _qR	ATTGCTGTTTGCTGCTGTC	49
S assimilation	*SULTR1;2*	AT1G78000	SULTR1;2_qF	GGATCCAGAGATGGCTACATGA	SULTR1;2_qR	TCGATGTCCGTAACAGGTGAC	50
	APR_2	AT1G62180	APR_2_qf	GCTTCAACTCTAATTGCTCCTGAAG	APR_2_qR	CAATGCAACATCTTCAGCTCCACT	
	APR_3	AT4G21990	APR_3_qF	GGAATCCATTGTTGCTTCTGAGGT	APR_3_qR	CAGAGCAACATCTTCAGCTCCACT	

Supplemental Table S4 Salt composition of Hoagland's medium

Component	Concentration of stock solution (mM)	Grams of salts (g)	Volume of stock solution per liter (ml)
KNO_3	1000	101.1	6
$Ca(NO_3)_2 \cdot 4H_2O$	1000	236.16	4
$NH_4H_2PO_4$	1000	115.08	2
$MgSO_4 \cdot 7H_2O$	1000	246.48	1
KCl	25	1.864	2
H_3BO_3	12.5	0.773	2
$MnSO_4 \cdot H_2O$	1	0.169	2
$ZnSO_4 \cdot 7H_2O$	1	0.288	2
CuSO4·5H2O	0.25	0.062	2
H_2MoO_4(85% MoO_3)	0.25	0.04	2
*Na·Fe·DTPA (10% Fe)	53.7	30	0.3-1

*Note: This should be avoided in salinity stress treatment imposition.
